# Non‐pharmacologic therapies for treating sexual dysfunction during pregnancy: A systematic review and meta‐analysis

**DOI:** 10.1002/ijgo.70451

**Published:** 2025-08-27

**Authors:** Antonio Carlos Queiroz de Aquino, Ayane Cristine Alves Sarmento, Ana Carolina Zimmermann Simões, Heitor Dutra de Medeiros, Beatriz Bomtempo de Siqueira, Tâmilly Batista Nascimento, Cijara Leonice de Freitas, Megan L. Falsetta, Ana Katherine Gonçalves

**Affiliations:** ^1^ Health Sciences Postgraduate Program Federal University of Rio Grande do Norte (UFRN) Natal Rio Grande do Norte Brazil; ^2^ Faculty of Health Sciences of Trairi Federal University of Rio Grande do Norte Santa Cruz Brazil; ^3^ Department of Clinical and Toxicological Analysis Federal University of Rio Grande do Norte (UFRN) RN Brazil; ^4^ Institute of Teaching, Research and Innovation League against Cancer Natal RN Brazil; ^5^ Sciences Applied to Women's Health Postgraduate Program Federal University of Rio Grande do Norte (UFRN) Natal RN Brazil; ^6^ School of Health Federal University of Rio Grande do Norte (UFRN) Natal Rio Grande do Norte Brazil; ^7^ Departments of Obstetrics and Gynecology and Pharmacology and Physiology University of Rochester Medical Center Rochester New York USA; ^8^ Department of Obstetrics and Gynecology Federal University of Rio Grande do Norte (UFRN) Natal Rio Grande do Norte Brazil

**Keywords:** meta‐analysis, pregnancy, sexual dysfunction, systematic review, therapeutics

## Abstract

**Background:**

Sexual dysfunction during pregnancy is a common problem, and can occur as the result of various physical, hormonal, and emotional changes that women experience during this period. Non‐pharmacologic treatments are recommended due to the restrictions on medications during this period.

**Objectives:**

To summarize the evidence on non‐pharmacologic interventions for treating sexual dysfunction during pregnancy.

**Search Strategy:**

Searches were conducted in PubMed, Scopus, Web of Science, Embase, PsycINFO, PEDro, the Cochrane Central Register of Controlled Trials, and ClinicalTrials.gov.

**Selection Criteria:**

We included randomized clinical trials comparing interventions for treating sexual dysfunction during pregnancy.

**Data Collection and Analysis:**

From these search results, studies were selected, and data was extracted by two authors independently. The risk of bias was assessed using the Cochrane Risk of Bias tool (RoB 2.0). RevMan 5.4. was used for data synthesis. The Grading of Recommendations Assessment Development (GRADE) and Evaluation method was used to evaluate the strength of the evidence.

**Main Results:**

We retrieved a total of 9017 articles. Twenty‐four studies met the eligibility criteria and were included in the systematic review, and nine studies were included in the meta‐analysis. The total number of participants was 1557, with a mean age ranging from 19.3 to 30.7 years and a gestation ranging from 8.9 to 28.3 weeks. Patients undergoing cognitive behavioral therapy (CBT) had a mean increase of 6.82 in their Female Sexual Function Index (FSFI) total score (range 1.63–12.01, *P* = 0.010, *I* ^2^ = 3%). For the Permission, Limited Information, Specific Suggestions, and Intensive Therapy (PLISSIT) model, the mean total FSFI score was 6.07 points higher (range 3.80–8.35, *P* = 0.00001, *I* ^2^ = 80%), and for the sex education intervention, the mean total score was 5.82 points higher (range 4.19–7.46, *P* = 0.00001, *I* ^2^ = 81%) than the control group.

**Conclusion:**

CBT, the PLISSIT model, and sexual education for pregnant women can improve sexual function during the gestational period.

## 1 INTRODUCTION

Sexual dysfunction during pregnancy is a complex and significant concern within the field of gynecology. It encompasses a spectrum of challenges that impact women's sexual health and intimate relationships during gestation. This phase marks a time of profound physical, hormonal, and emotional changes, which inevitably influence a woman's sexual experience.^1^, ^2^, ^3^, ^4^

Throughout pregnancy, various factors contribute to sexual dysfunction. These include physical changes such as weight gain, breast enlargement, and a growing abdomen, which can lead to altered body image, reduced self‐esteem, and decreased comfort with intimacy.^5^, ^6^, ^7^ Hormonal fluctuations also play a crucial role. Elevated levels of estrogen, progesterone, and prolactin can affect vaginal lubrication, potentially causing discomfort or pain during sexual intercourse. Additionally, psychological factors such as anxiety, fears about potential harm to the fetus or oneself during sexual activity, and concerns about childbirth may contribute to sexual dysfunction.^3^, ^5^, ^6^, ^7^ The prevalence and nature of sexual dysfunction during pregnancy can vary widely among individuals. Some women experience an increase in sexual desire and satisfaction, whereas others encounter challenges such as decreased libido, difficulty achieving orgasm, or pain during intercourse (dyspareunia).^8^

Unfortunately, sexual health during pregnancy often remains under‐addressed in clinical settings. Many healthcare providers tend to focus primarily on the physical aspects of pregnancy, neglecting the emotional and sexual needs of expectant mothers.^7^ Open communication between healthcare providers and expectant mothers is essential to address concerns, provide reassurance, and offer guidance for maintaining a healthy sexual relationship during pregnancy.^9^

Research in this area is crucial to better understand the factors contributing to sexual dysfunction during pregnancy and to develop effective interventions that support sexual health and well‐being for expectant mothers and their partners. Clinical trials involving non‐pharmacologic interventions, such as behavioral therapies, counseling, and specific exercises, are being studied to treat sexual dysfunction during this stage of a woman's life.^10^ Considering the multitude of factors associated with sexual dysfunction and the limitations of pharmacologic interventions during pregnancy, including limited safety data, this study aims to systematically evaluate the efficacy of non‐pharmacologic interventions for treating sexual dysfunction during pregnancy.

## 2 MATERIALS AND METHODS

This review follows the Preferred Reporting Items for Systematic Reviews and Meta‐Analyses (PRISMA) guidelines.^11^, ^12^, ^13^ The protocol is registered with the International Prospective Register of Systematic Reviews (PROSPERO CRD42022382974).

### 2.1 Ethical considerations

Secondary data were used in this study, so obtaining approval from the ethics committee was not necessary.

### 2.2 Search strategy

The search in the databases was carried out under the guidance of an experienced librarian from the Center for Health Sciences (CHS—UFRN, Natal, Brazil). PubMed, Scopus, Web of Science, Embase, PsycINFO, PEDro, Cochrane Central Register of Controlled Trials, and ClinicalTrials.gov were searched with no limitations on publication date or language. All electronic databases were searched on July 3, 2024. The detailed search strategy for each database is described in Table 1.

**TABLE 1:** Detailed search strategy for each database.

Database	Search strategy
Cochrane, Medline/PubMed and Web of Science	(Pregnancy OR Gestation OR Pregnant Women OR Women, Pregnant) AND (Therapeutic OR Therapeutics OR Therapy OR Therapies OR Treatment OR non‐pharmacological treatment) AND (Sexual Dysfunctions, Psychological OR Physiological Sexual Dysfunction OR Dysfunction, Psychological Sexual OR Sexual Arousal Disorder OR Sexual satisfaction OR Sexual Dysfunction, Physiological OR Sex Disorders OR Sexual Distress OR Body Image)
Scopus	Pregnancy OR Gestation OR “Pregnant Women” OR “Women, Pregnant” AND Therapeutic OR Therapeutics OR Therapy OR Therapies OR Treatment OR “non‐pharmacological treatment” AND “Sexual Dysfunctions, Psychological” OR “Physiological Sexual Dysfunction” OR “Dysfunction, Psychological Sexual” OR “Sexual Arousal Disorder” OR “Sexual satisfaction” OR “Sexual Dysfunction, Physiological” OR “Sex Disorders” OR “Sexual Distress” OR “Body Image”
Embase	(Pregnancy OR Gestation OR Pregnant Women OR Women, Pregnant) AND (Therapeutic OR Therapeutics OR Therapy OR Therapies OR Treatment OR non‐pharmacological treatment) AND (Sexual Dysfunctions, Psychological OR Physiological Sexual Dysfunction OR Dysfunction, Psychological Sexual OR Sexual Arousal Disorder OR Sexual satisfaction OR Sexual Dysfunction, Physiological OR Sex Disorders OR Sexual Distress OR Body Image) + FILTER DISEASES “SEXUAL DYSFUNCTION”
PEDro, PsycINFO and clinicaltrials.gov	“Sexual Dysfunction” AND (Pregnancy OR gestation OR “Pregnant women”)

### 2.3 Eligibility criteria

Randomized clinical trials (RCTs) that compared interventions for treating sexual dysfunction during pregnancy were included. Observational studies, case reports, review articles, reports, and case series were excluded.

### 2.4 PICOT strategy

The PICOT strategy followed was as follows. Population/Participants: pregnant women with sexual dysfunction; Intervention: any therapy used to treat pregnant women with sexual dysfunction; Comparator/control: placebo or other treatment; Outcomes: improvement of sexual dysfunction; and Type of study: randomized clinical trials.

### 2.5 Selection process

The articles were imported to Rayyan (Mourad Ouzzani, University of Oxford, UK), and duplicates were removed. Three authors independently (ACQA, TBN and BBS) screened by title, abstract, and full text to determine inclusion criteria. A fourth author (AKG) resolved any discrepancies.

### 2.6 Primary outcome

Our primary outcome was improvement of sexual function as assessed by validated questionnaires, scales, or tools, either by subdomains or by total score.

### 2.7 Data extraction

Two authors (HDM and ACZS) independently extracted data from the included studies. Disagreements were resolved with discussion between the authors. The data were collected in standardized Excel forms. We also extracted the following data: author, year, country, number of participants, gestational age (weeks), mean age (years), groups intervention, instrument measurements, follow up (weeks), and relevant results.

### 2.8 Synthesis methods

A meta‐analysis was performed if a minimum of two studies assessed a similar population using the same outcome(s) and tool(s) (e.g. sexual function evaluated by Female Sexual Function Index [FSFI]). The software Review Manager (RevMan) V.5.4.1 was used to perform the meta‐analysis. The mean difference with 95% confidence interval (CI) was calculated by continuous data (mean difference and SD) to obtain a summary of the overall estimate. Data were pooled using a random‐effects model. Heterogeneity was assessed using the *I* ^2^ statistic was interpreted as low (<50%), moderate (50%–75%), or high (>75%).^14^

### 2.9 Missing data

In the case of a lack of data (incomplete studies or missing values/measures), the authors or co‐authors of the article were contacted by email. When the missing information was not received, the data were excluded from analysis and are mentioned in the Discussion section.

### 2.10 Sensitivity analyses

We performed sensitivity analyses to assess the robustness of the studies included in the meta‐analysis. Initially, we conducted a meta‐analysis encompassing all studies. Subsequently, we refined our analysis by including only those studies deemed definitively eligible or those with a low risk of bias.^14^

### 2.11 Quality assessment

Two authors (ACQA and CLF) independently assessed the risk of bias using the Cochrane Risk of Bias Tool (RoB 2).^15^ Disagreements were resolved with discussion between the authors. In cases where an agreement could not be reached, a third author (ACAS), “broke the tie.” In each study, we evaluated the following: the randomization process, deviations from intended interventions, missing outcome data, measurement of the outcome, and selection of the reported results.

### 2.12 Assessing certainty in the findings

The quality of evidence was assessed by two authors independently (ACQA and ACAS). The Grading of Recommendations Assessment Development and Evaluation (GRADE) approach was used to evaluate the strength of the evidence of the systematic review results.^16^

## 3 RESULTS

### 3.1 Study selection

**FIGURE 1:**
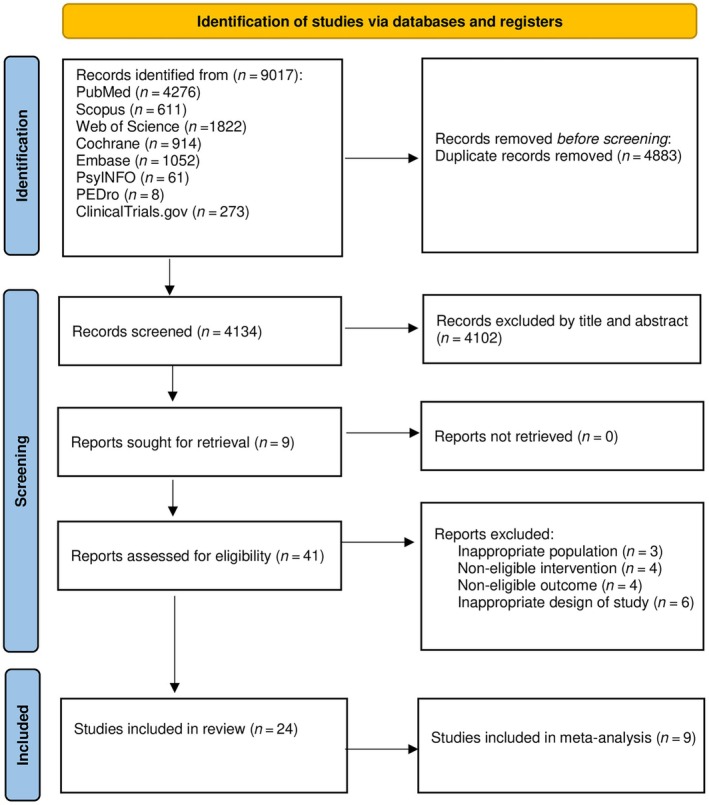
PRISMA flow chart.

The database search retrieved 9017 articles, of which 4883 were duplicates and were removed. Of these, after reading the title and abstract, 4102 were excluded. After reading the full text, 24 studies were included in the systematic review.^17^, ^18^, ^19^, ^20^, ^21^, ^22^, ^23^, ^24^, ^25^, ^26^, ^27^, ^28^, ^29^, ^30^, ^31^, ^32^, ^33^, ^34^, ^35^, ^36^, ^37^, ^38^, ^39^, ^40^ These studies included a total of 1557 participants. Nine studies could be combined in a meta‐analysis.^17^, ^23^, ^25^, ^26^, ^27^, ^31^, ^36^, ^37^, ^40^ The PRISMA flow chart summarizes the selection process (Figure 1).

### 3.2 Study characteristics

The studies analyzed were published between 2010 and 2023. Among the articles included in the review, 21 originated from Iran, two from Turkey, and one from Thailand. The mean age of participants ranged from 19.3 to 30.7 years, and gestational age ranged from 8.9 to 28.3 weeks. The interventions carried out were cognitive behavioral therapy (CBT), Sex Education, Permission, Limited Information, Specific Suggestions and Intensive Therapy (PLISSIT), pelvic floor muscle exercises (PFME), Kegel exercises, mindfulness, and yoga. For the comparison group, study controls received routine consultations only. The assessment instruments were: the FSFI, Pregnancy Sexual Response Inventory (PSRI), the Female Sexual Quality of Life (SQL‐F), the Larson Sexual Satisfaction Questionnaire (LSSQ), the Sexual Beliefs Questionnaire (SBQ), the Married Female Sexual Satisfaction Questionnaire (MWSSQ), the Linda Berg Sexual Satisfaction Scale (LBSSS), visual analog scale (VAS), the Female Sexual Distress Scale‐Revised (FSDS‐R), and the Sexual Satisfaction Scale for Women (SSSW). Follow‐up lasted between 2 and 8 weeks. The summary of these data is described in Table 2.

**TABLE 2:** Summary of findings of the included randomized controlled trials.

First author (year), Country	Number of participants	Gestational age, weeks	Mean age, years	Groups	Instrument measures	Follow up, weeks	Relevant results
Afshar (2012)^17^ Iran	IG: 41 CG: 42	IG: 8.9 CG: 9.3	IG: 26.6 CG: 26.7	IG: Sexual education CG: Routine consult	FSFI	4	There was a significant difference in all six domains of sexual function (desire, arousal, lubrication, orgasm, satisfaction, and pain) assessed by the FSFI (*P* < 0.001)
Alizadeh (2021)^18^ Iran	IG1: 50 IG2: 53 CG: 51	IG1: ND IG2: ND CG: ND	IG1: 28.3 IG2: 28.6 CG: 29.3	IG1: Sexual education IG2: Sexual education self‐training CG: Routine consult	PSRI and SQOL‐F	4	The mean PSRI and SQOL‐F scores in the training group (IG1) increased from early to late pregnancy compared with the CG and self‐training group (IG2) (*P* < 0.001)
Amini (2021)^19^ Iran	IG: 20 CG: 20	IG: ND CG: ND	IG: 29.0 CG: 29.4	IG: CBT CG: Routine consult	FSFI	8	There was a significant difference in all subdomains of the FSFI (*P* < 0.05) for CBT
Barvanloo‐Golmohamadi (2022)^20^ Iran	IG: 30 CG: 31	IG: 22.8 CG: 2.4	IG: 27.6 CG: 30.2	IG: Sexual education CG: Routine consult	LSSQ	8	Comparison of differences between the sexual satisfaction scores before and after the intervention indicated a significant change (*P* = 0.009). This change was not significant in the CG (*P* = 0.46)
Bokaie (2022)^21^ Iran	IG: 26 CG: 26	IG: ND CG: ND	IG: 26.2 CG: 26.5	IG: CBT CG: Routine consult	SBQ and LSSQ	4	For SBQ, the mean sexual satisfaction score was significantly higher 1 month after CBT (*P* < 0.001). For LSSQ, the mean sexual satisfaction score was significantly higher 1 month after CBT (*P =* 0.04). In the CG, there were no significant differences between the mean sexual satisfaction scores before and immediately after the intervention (*P* = 0.381)
Cengizhan (2023)^22^ Turkey	IG: 67 CG: 67	IG: ND CG: ND	IG: 28.6 CG: 29.7	IG: Mindfulness CG: Routine consult	FSDS‐R	4	Mean scores for sexual distress decreased significantly in the mindfulness group compared to the CG (*P* < 0.001)
Fathalian (2022)^23^ Iran	IG: 40 CG: 40	IG: 20.3 CG: 20.1	IG: 26.0 CG: 26.5	IG: CBT CG: Routine consult	FSFI	4	The average sexual function scores in the IG increased significantly compared to the CG (*P* < 0.001)
Ghorbanzade (2019)^24^ Iran	IG: 32 CG: 33	IG: 21.1 CG: 22.3	IG: 21.2 CG: 21.4	IG: Kegel exercises CG: Routine consult	FSFI	6	There was a significant difference between the mean satisfaction and sexual function scores after the intervention (*P* < 0.001)
Heidari^(a)^ (2017)^25^ Iran	IG: 42 CG: 41	IG: ND CG: ND	IG: ND CG: ND	IG: Sexual education CG: Routine consult	FSFI	4	Repeated measures analysis showed significant differences between groups in mean total FSFI scores during the third trimester (*P* = 0.001)
Heidari^(b)^ (2017)^26^ Iran	IG: 42 CG: 41	IG: ND CG: ND	IG: 25.7 CG: 25.7	IG: PLISSIT CG: Routine consult	FSFI	4	Repeated measures analysis showed significant differences between the two groups in terms of mean FSFI total scores post‐intervention (*P* < 0.05)
Mahnaz (2019)^27^ Iran	IG: 36 CG: 34	IG: ND CG: ND	IG: 26.3 CG: 26.7	IG: Sexual education CG: Routine consult	FSFI	4	The results for the total score and most FSFI subdomains (sexual desire, arousal, lubrication, orgasm, satisfaction) were significantly different (*P* < 0.001). However, there was no difference for the pain subdomain (*P* = 0.78) after the intervention compared with the CG
Masoumi (2018)^28^ Iran	IG: 30 CG: 30	IG: 28 CG: 27	IG: ND CG: ND	IG: Sexual education CG: Routine consult	LBSSS	4	Mean sexual satisfaction scores increased significantly after the sexual education intervention (*P* = 0.029)
Naji Abhary (2022)^29^ Iran	IG: 30 CG: 30	IG: 23.2 CG: 23.2	IG: 29.6 CG: 30.7	IG: Mindfulness CG: Routine consult	SSSW	4	After the intervention, the average sexual satisfaction score in pregnant women increased significantly compared with the CG (*P* < 0.001)
Navidian (2017)^30^ Iran	IG: 50 CG: 50	IG: 19.7 CG: 21.4	IG: 27.3 CG: 26.5	IG: Sexual education CG: Routine consult	PSRI	2	Sexual counseling improved sexual function for women in the IG as far as sexual desire, sexual frequency, satisfaction, arousal, orgasm, and sexual quality at post‐test (*P* < 0.01)
Nejati (2017)^31^ Iran	IG: 40 CG: 40	IG: ND CG: ND	IG: 26.3 CG: 27.1	IG: PLISSIT CG: Routine consult	FSFI	4	After adjusting for pre‐intervention scores, there was a significant difference between the FSFI mean sexual function score and all its subdomains between the IG and CG in the fourth week post‐intervention (*P* < 0.05)
Nejati (2020)^32^ Iran	IG: 40 CG: 40	IG: 24.7 CG: 24.7	IG: 26.3 CG: 27.1	IG: PLISSIT CG: Routine consult	LBSSS	4	There were significant differences between the mean sexual function scores and all subdomains, between the IG and CG 4 weeks post‐intervention (*P* < 0.05)
Nezamnia (2020)^33^ Iran	IG: 18 CG: 15	IG: 14.9 CG: 15.1	IG: 21.8 CG: 19.3	IG: CBT CG: Routine consult	FSFI	8	There was a significant difference in sexual function between the case and control groups 2 and 4 weeks after the intervention (*P* < 0.001)
Pourkhiz (2017)^34^ Iran	IG: 41 CG: 41	IG: 19.1 CG: 19.0	IG: 26.0 CG: 25.3	IG: PFME CG: Routine consult	FSFI	4	The mean total sexual function score was significantly higher in the PFME training group during pregnancy (*P* < 0.001)
Saniei (2022)^35^ Iran	IG: 32 CG: 33	IG: 19.9 CG: 18.9	IG: 25.2 CG: 25.0	IG: Mindfulness CG: Routine consult	FSFI	4	There was no significant difference between the mean pre‐ and post‐test sexual desire scores for either group (*P* > 0.05). However, the two groups were significantly different in terms of mean sexual satisfaction scores before and after the intervention (*P* < 0.05)
Shahbazi (2019)^36^ Iran	IG: 35 CG: 35	IG: ND CG: ND	IG: 25.7 CG: 27.5	IG: PLISSIT CG: Routine consult	FSFI	4	There was a significant difference between the mean FSFI total score and all subdomains for the intervention groups compared to the control four weeks post‐intervention (*P* < 0.05). PLISSIT significantly reduced the frequency of sexual dysfunction, and there was a significant difference between the two groups (*P* < 0.001)
Vakilian (2018)^37^ Iran	IG: 11 CG: 11	IG: 10.5 CG: 15.3	IG: 22.7 CG: 25.8	IG: CBT CG: Routine consult	FSFI	4	One week after CBT, average sexual function increased compared with the control group (*P* = 0.001). Three months after the counseling sessions, a significant difference was observed between the two groups (*P* = 0.001). After the educational sessions, the average sexual function index significantly increased in the intervention versus control group (*P* = 0.001)
Wannakosit (2010)^38^ Thailand	IG: 39 CG: 32	IG: 22.6 CG: 21.4	IG: 30.0 CG: 29.0	IG: Sexual education CG: Routine consult	VAS	6	There was an increase in the frequency of sexual intercourse in the sex education group (*P* < 0.05). There were no statistically significant changes in desire, arousal, satisfaction, or orgasm (*P* > 0.05)
Yildiz Karaahmet (2022)^39^ Turkey	IG: 71 CG: 69	IG: ND CG:ND	IG: 28.0 CG: 26.6	IG: Yoga CG: Routine consult	FSFI	4	The mean FSFI score in the yoga group was significantly higher in the post‐test compared to the pre‐test (*P* = 0.002). In the control group, there was no difference between the mean FSFI scores pre‐ and post‐test (*P* = 0.181)
Ziaei (2022)^40^ Iran	IG: 25 CG: 27	IG: ND CG: ND	IG: 25.5 CG: 25.3	IG: PLISSIT CG: Routine consult	FSFI	4	There was a statistically significant difference in the mean sexual satisfaction scores between the groups during the follow‐up period (*P* = 0.01)

Abbreviations: CBT, cognitive behavioral therapy; CG, control group; FSFI, Female Sexual Function Index; FSDS‐R, Female Sexual Distress Scale‐Revised; IG, intervention group; LBSSS, Linda Berg Sexual Satisfaction Scale; LSSQ, Larson's Sexual Satisfaction Questionnaire; MWSSQ, Married Women's Sexual Satisfaction Questionnaire; ND, not described; PFME, pelvic floor muscle exercises; PSRI, Pregnancy Sexual Response Inventory; SBQ, Sexual Beliefs Questionnaire; SQL‐F, Sexual Quality of Life‐Female; SSSW, Sexual Satisfaction Scale for Women; VAS, visual analog scale.

Several articles could not be combined in the meta‐analysis, so we have summarized their findings here. Studies that evaluated sexual education compared with the control group used different scales and measurements of sexual function, making it impossible to directly compare results between studies. In Alizadeh et al.,^18^ mean PSRI scores in the sex education training group increased from early to late pregnancy compared with the control group (*P* < 0.001). Navidian et al.,^30^ using the same scale, showed that sexual education improved women's sexual function in terms of sexual desire, sexual frequency, satisfaction, arousal, orgasm, and sexual quality (*P* < 0.01).

Only Barvanloo‐Golmohamadi et al.^20^ used the LSSQ tool, and their results showed differences between sexual satisfaction scores before and after the intervention (sexual education) (*P* = 0.009). Using the LBSSS tool, Masoumi et al.,^28^ demonstrated that average sexual satisfaction scores increased significantly after sexual education compared with the control group that did not receive this education (*P* = 0.029). Wannakosit and Phupong,^38^ using the VAS tool, demonstrated that there was an increase in the frequency of sexual intercourse in the sexual education group (*P* < 0.05). However, there were no statistically significant changes in desire, excitement, satisfaction, or orgasm (*P* > 0.05).

Two studies that evaluated the CBT intervention in comparison to the control group (routine consultation) used two different tools. Amini et al.^19^ showed that there was a significant difference in all FSFI domains (*P* < 0.05) for CBT. Nezamnia et al.^33^ also showed a significant difference in sexual function using FSFI between the intervention and control groups after the intervention (*P* < 0.001).

One study assessed sexual belief and sexual satisfaction using the SBQ and LSSQ tools, respectively. For SBQ, the mean sexual satisfaction score was significantly higher 1 month after CBT (*P* < 0.001). For LSSQ, the mean sexual satisfaction score was significantly higher 1 month after CBT (*P* = 0.04).^21^

The studies that evaluated mindfulness used three different tools. Cengizhan and Uçar^22^ used the FSDS‐R Tool and observed that mean sexual distress scores decreased in the mindfulness group compared with the control group (*P* < 0.001). Naji Abhary et al.^29^ used the SSSW tool and showed that the average sexual satisfaction score in pregnant women increased compared with the control group (*P* < 0.001). Finally, Saniei et al.^35^ showed that the mindfulness and control groups were significantly different in terms of mean sexual satisfaction FSFI scores before and after the intervention (*P* < 0.05).

Various interventions have some effect on sexual function. For the intervention of Kegel exercises, there was a significant difference between the mean FSFI scores in satisfaction and sexual function compared with the control group (*P* < 0.001).^24^ In the studies using PLISSIT intervention, there were significant differences between the mean sexual function score and all its domains endorsed by the LBSSS tool, compared with the control group (*P* < 0.05).^32^ In an RCT that evaluated the PFME, the mean total sexual function score assessed by the FSFI was significantly higher compared with the control group (*P* < 0.001).^34^ Finally, in a study that evaluated yoga, the average FSFI score in the intervention group was significantly higher (*P* = 0.002). In the control group, there was no difference between the mean FSFI scores (*P* = 0.181).^39^

Of the articles eligible for meta‐analysis, four studies evaluated the effect of the PLISSIT model on sexual function,^26^, ^31^, ^36^, ^40^ two studies evaluated CBT,^23^, ^37^ and three studies analyzed sexual education.^17^, ^25^, ^27^

### 3.3 Synthesis of results

**FIGURE 2:**
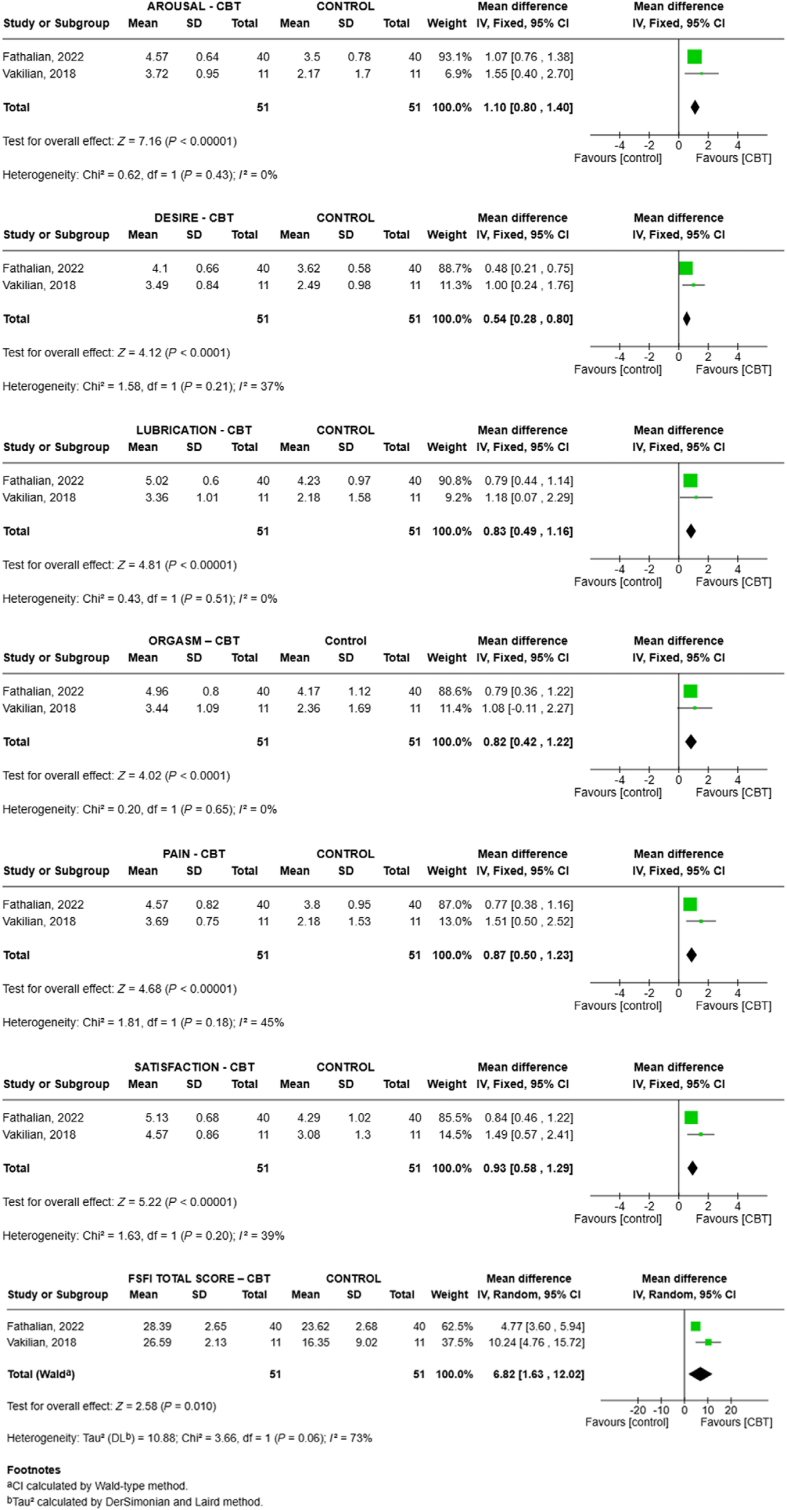
Meta‐analysis comparing the effectiveness of the cognitive behavioral therapy (CBT) intervention versus control for all domains and total score of the Femal Sexual Function Index (FSFI).

For CBT, FSFI domain scores increased on average (vs. control) as follows (Figure 2): desire 0.54 (range 0.28–0.80, *P* < 0.001, *I* ^2^ = 37%), arousal 1.10 (range 0.80–1.40, *P* < 0.001, *I* ^2^ = 0%), lubrication 0.83 (range 0.49–1.16, *P* < 0.001, *I* ^2^ = 0%), orgasm 0.82 (range 0.42–1.22, *P* < 0.001, *I* ^2^ = 0%), satisfaction 0.93 (range 0.58–1.29, *P* < 0.001, *I* ^2^ = 39%), pain 0.87 (range 0.50–1.23, *P* < 0.001, *I* ^2^ = 45%), and total score 6.82 (range 1.63–12.01, *P* = 0.010, *I* ^2^ = 73%) (Figure 2).

**FIGURE 3:**
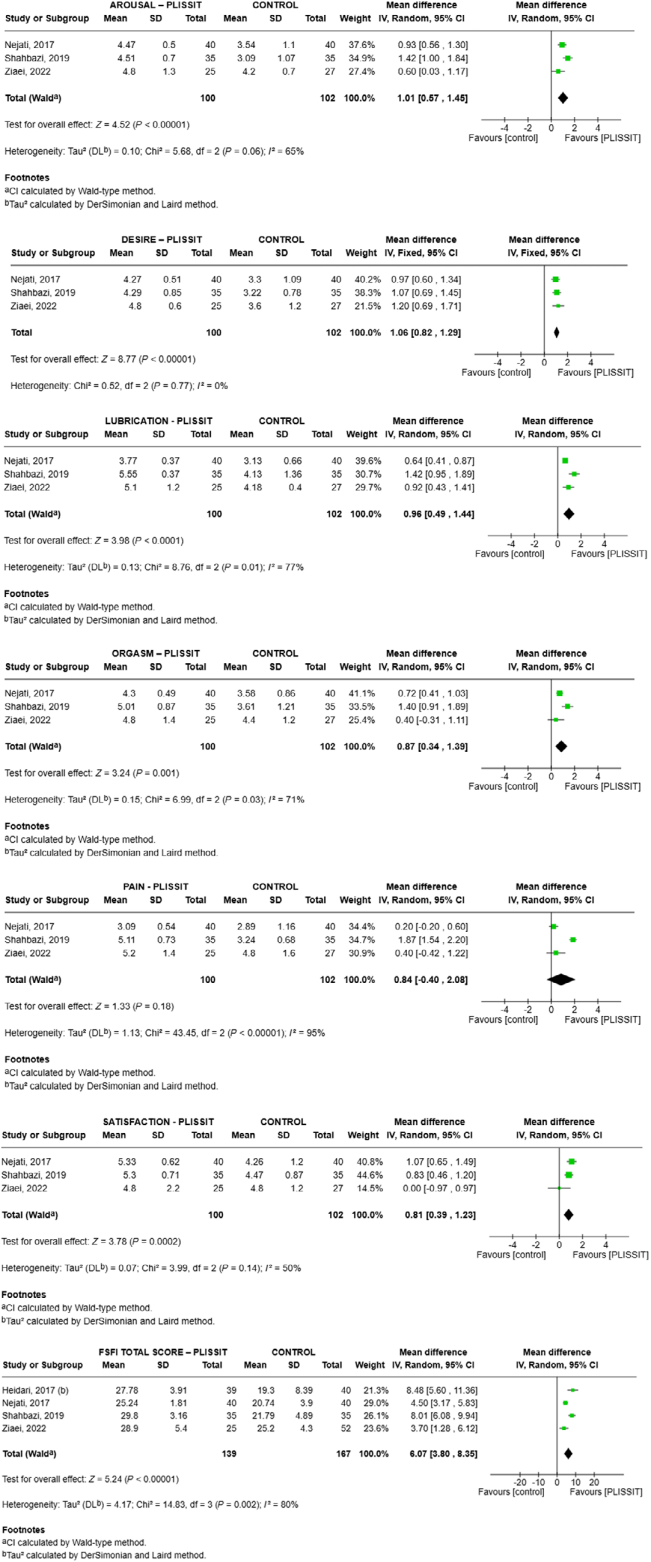
Meta‐analysis comparing the effectiveness of the PLISSIT intervention versus control for all domains and total score of the Femal Sexual Function Index (FSFI).

For PLISSIT, FSFI domain scores increased on average (vs. control) as follows (Figure 3): desire 1.06 (range 0.82–1.29, *P* < 0.001, *I* ^2^ = 0%), arousal 1.01 (range 0.57–1.45, *P* < 0.001, *I* ^2^ = 65%), lubrication 0.96 (range 0.49–1.44, *P* < 0.001, *I* ^2^ = 77%), orgasm 0.87 (range 0.34–1.39, *P* = 0.001, *I* ^2^ = 71%), satisfaction 0.81 (range 0.39–1.23, *P* < 0.001, *I* ^2^ = 50%), pain 0.84 (range −0.40 to 2.08, *P* = 0.18, *I* ^2^ = 95%), and total score 6.07 (range 3.80–8.35, *P* < 0.001, *I* ^2^ = 80%) (Figure 3).

**FIGURE 4:**
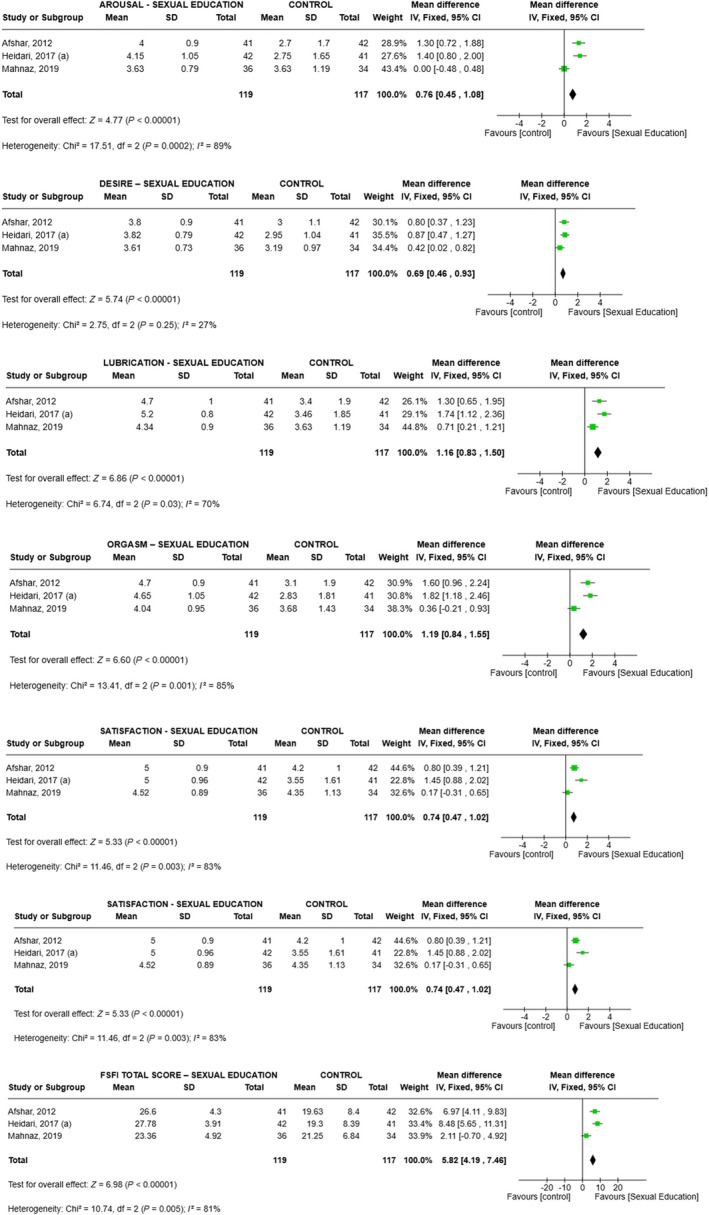
Meta‐analysis comparing the effectiveness of the Sexual Education intervention versus control for all domains and total score of the Femal Sexual Function Index (FSFI).

Finally, for sex education, FSFI domain scores increased on average (vs. control) as follows (Figure 4): desire 0.69 (range 0.46–0.93, *P* < 0.001, *I* ^2^ = 27%), arousal 0.76 (range 0.45–1.08, *P* < 0.001, *I* ^2^ = 89%), lubrication 1.16 (range 0.83–1.50, *P* < 0.001, *I* ^2^ = 70%), orgasm 1.19 (range 0.84–1.55, *P* < 0.001, *I* ^2^ = 85%), satisfaction 0.74 (range 0.47–1.02, *P* < 0.001, *I* ^2^ = 83%), pain 0.56 (range 0.27–0.86, *P* < 0.001, *I* ^2^ = 91%), and total score 5.82 (range 4.19–7.46, *P* < 0.001, *I* ^2^ = 81%) (Figure 4).

### 3.4 Risk of bias of included studies

**FIGURE 5:**
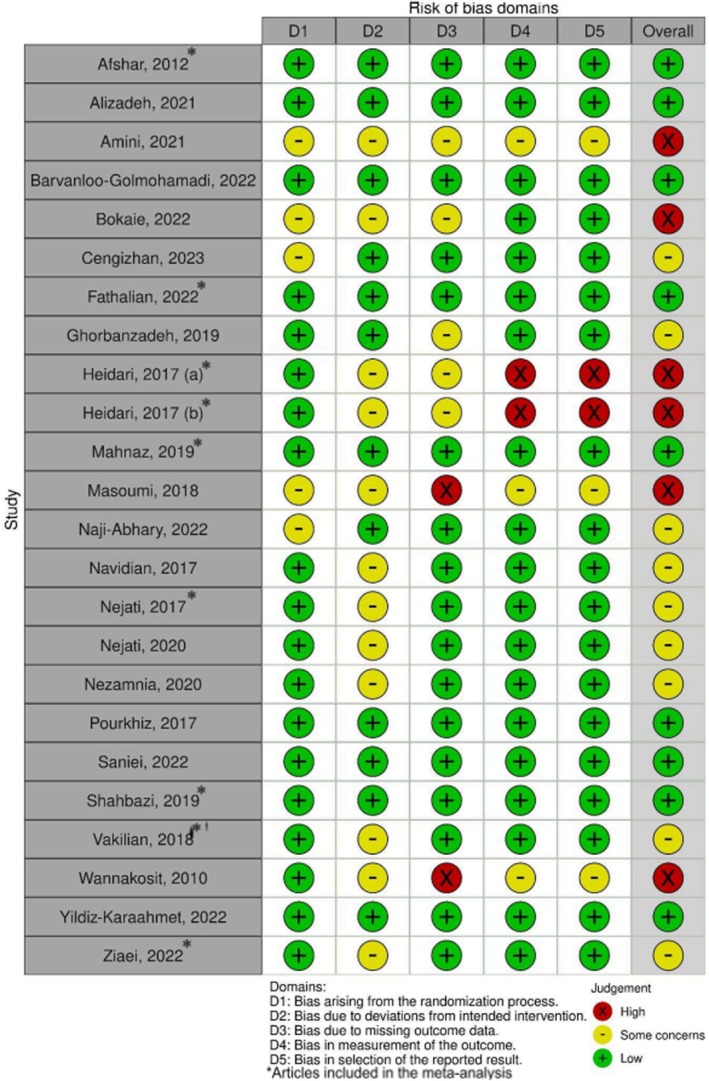
Risk of bias of the included studies.

Nine studies were considered low risk.^17^, ^18^, ^20^, ^23^, ^27^, ^34^, ^35^, ^36^, ^39^ Nine studies were of concern,^22^, ^24^, ^29^, ^30^, ^31^, ^32^, ^33^, ^37^, ^40^ because they did not report in detail their blinding processes. There were also deviations from intended interventions or outcome data were missing outcome. Six were high risk,^19^, ^21^, ^25^, ^26^, ^28^, ^38^ due to missing outcome data, incorrect measurement of results, and inappropriate selection of reported results. The risk of bias assessment for each study is shown in Figure 5.

### 3.5 Quality of evidence

The GRADE rating for the certainty of the evidence for improvement of sexual function using CBT was considered high for all outcomes evaluated by FSFI. For the PLISSIT intervention, it was considered high for all domains, except for the total score (very low). Finally, for the sexual education intervention, the certainty of evidence was moderate for desire, low for arousal, lubrication, orgasm and satisfaction, and very low for pain and total score because of the high heterogeneity, large confidence interval ranges, and bias in the measurement of the outcomes and selection of the reported results (Table 3).

**TABLE 3:** GRADE assessment.

Certainty assessment							No. of patients		Effect		Certainty	Importance
No. of studies	Study design	Risk of bias	Inconsistency	Indirectness	Imprecision	Other considerations	PLISSIT	Control	Relative (95% CI)	Absolute (95% CI)		
Desire												
3	Randomized trials	Not serious	Not serious	Not serious	Not serious^a^	None	100	102	—	MD **1.06 higher** (0.82 higher to 1.29 higher)	⨁⨁⨁⨁ High	IMPORTANT
Arousal												
3	Randomized trials	Not serious	Not serious	Not serious	Not serious^a^	None	100	102	—	MD **1.01 higher** (0.57 higher to 1.45 higher)	⨁⨁⨁⨁ High	IMPORTANT
Lubrication												
3	Randomized trials	Not serious	Not serious	Not serious	Not serious^a^	None	100	102	—	MD **0.96 higher** (0.49 higher to 1.44 higher)	⨁⨁⨁⨁ High	IMPORTANT
Orgasm												
3	Randomized trials	Not serious	Not serious	Not serious	Not serious^a^	None	100	102	—	MD **0.87 higher** (0.34 higher to 1.39 higher)	⨁⨁⨁⨁ High	IMPORTANT
Satisfaction												
3	Randomized trials	not serious	Not serious	Not serious	Not serious^a^	None	100	102	—	MD **0.81 higher** (0.39 higher to 1.23 higher)	⨁⨁⨁⨁ High	CRITICAL
Pain												
3	Randomized trials	Not serious	Not serious	Not serious	Not serious^a^	None	100	102	—	MD **0.84 higher** (0.4 lower to 2.08 higher)	⨁⨁⨁⨁ High	CRITICAL
Total score												
4	Randomized trials	Not serious	Very serious^b^	Serious^c^	Not serious	None	139	167	—	MD **6.07 higher** (3.8 higher to 8.35 higher)	⨁◯◯◯ Very low	CRITICAL

Certainty assessment							No. of patients		Effect		Certainty	Importance
No. of studies	Study design	Risk of bias	Inconsistency	Indirectness	Imprecision	Other considerations	Sexual Education	Control	Relative (95% CI)	Absolute (95% CI)		
Desire												
3	Randomized trials	Serious^a^	Not serious	Not serious	Not serious	None	119	117	—	MD **0.69 higher** (0.46 higher to 0.93 higher)	⨁⨁⨁◯ Moderate	IMPORTANT
Arousal												
3	Randomized trials	Serious^a^	Serious^b^	Not serious	Not serious	None	119	117	—	MD **76 higher** (0.45 higher to 1.08 higher)	⨁⨁◯◯ Low	IMPORTANT
Lubrication												
3	Randomized trials	Serious^a^	Serious^c^	Not serious	Not serious	None	119	117	—	MD **1.16 higher** (0.83 higher to 1.5 higher)	⨁⨁◯◯ Low	IMPORTANT
Orgasm												
3	Randomized trials	Serious^a^	Serious^d^	Serious^e^	Not serious	None	119	117	—	MD **1.19 higher** (0.84 higher to 1.55 higher)	⨁◯◯◯ Very low	IMPORTANT
Satisfaction												
3	Randomized trials	Serious^a^	Serious^f^	Not serious	Not serious	None	119	117	—	MD **0.74 higher** (0.47 higher to 1.02 higher)	⨁⨁◯◯ Low	CRITICAL
Pain												
3	Randomized trials	Serious^a^	Serious^g^	Serious^e^	Not serious	None	119	117	—	MD **0.56 higher** (0.27 higher to 0.86 higher)	⨁◯◯◯ Very low	CRITICAL
Total score												
3	Randomized trials	Serious^a^	Serious^h^	Very serious^e^	Not serious	None	119	117	—	MD **5.82 higher** (4.19 higher to 7.46 higher)	⨁◯◯◯ Very low	CRITICAL

Certainty assessment							No. of patients		Effect		Certainty	Importance
No. of studies	Study design	Risk of bias	Inconsistency	Indirectness	Imprecision	Other considerations	CBT	Control	Relative (95% CI)	Absolute (95% CI)		
Desire												
2	Randomized trials	Not serious	Not serious	Not serious	Not serious^a^	None	51	51	—	MD **0.54 higher** (0.28 higher to 0.8 higher)	⨁⨁⨁⨁ High	IMPORTANT
Arousal												
2	Randomized trials	Not serious	Not serious^b^	Not serious^c^	Not serious^a^	None	51	51	—	MD **1.1 higher** (0.8 higher to 1.4 higher)	⨁⨁⨁⨁ High	IMPORTANT
Lubrication												
2	Randomized trials	Not serious	Not serious^d^	Not serious^c^	Not serious^a^	None	51	51	—	MD **0.83 higher** (0.49 higher to 1.16 higher)	⨁⨁⨁⨁ High	IMPORTANT
Orgasm												
2	Randomized trials	Not serious	Not serious^e^	Not serious^c^	Not serious^a^	None	51	51	—	MD **0.82 higher** (0.42 higher to 1.22 higher)	⨁⨁⨁⨁ High	IMPORTANT
Satisfaction												
2	Randomized trials	Not serious	Not serious^e^	Not serious^c^	Not serious^a^	None	51	51	—	MD **0.93 higher** (0.58 higher to 1.29 higher)	⨁⨁⨁⨁ High	CRITICAL
Pain												
2	Randomized trials	Not serious	Not serious^b^	Not serious^c^	Not serious^a^	None	51	51	—	MD **0.87 higher** (0.5 higher to 1.23 higher)	⨁⨁⨁⨁ High	CRITICAL
Total score												
2	Randomized trials	Not serious	Not serious^f^	Not serious^c^	Not serious	None	51	51	—	MD **6.82 higher** (1.63 higher to 12.01 higher)	⨁⨁⨁⨁ High	CRITICAL

*Note*: Bold values indicates number of Participants.

Abbreviations: CBT, cognitive behavioral therapy; CI, confidence interval; MD, mean difference.

^a^ Few events.

^b^ High heterogeneity.

^c^ Large confidence interval.

^d^ High heterogeneity (*I*²= 85%).

^e^ Large confidence interval.

^f^ High heterogeneity (*I*² = 83%).

^g^ High heterogeneity (*I*² = 91%).

^h^ High heterogeneity (*I*² = 81%).

## 4 DISCUSSION

### 4.1 Principal findings

This systematic review and meta‐analysis evaluated all available clinical trials addressing non‐pharmacologic therapies for treating sexual dysfunction in pregnant women.

The three different interventions discussed in the meta‐analysis showed improvement in sexual function across almost all domains of the FSFI. For CBT^23^, ^37^ and sexual education,^17^, ^25^, ^27^ improvements were seen in all domains: desire, arousal, lubrication, orgasm, satisfaction, pain, and total score. For the PLISSIT model,^26^, ^31^, ^36^, ^40^ improvements were noted in the domains of desire, arousal, lubrication, orgasm, satisfaction, and total score, but not in the pain domain.

When assessing the certainty of evidence, we found that the certainty regarding improvement in sexual function through CBT and the PLISSIT model was consistently high for nearly all domains. Conversely, for the sexual education intervention, the quality of evidence varied between moderate, low, and very low. The variations in the certainty of evidence for the total PLISSIT score, as assessed by GRADE, can be attributed to the high heterogeneity of data across the studies and the wide confidence intervals obtained in the meta‐analysis. Additionally, the risk of bias in most studies raised concerns, primarily because of the lack of participant blinding inherent in the nature of the interventions.

It is important to note that the majority of studies in this review were conducted in predominantly Muslim and partly Arab regions (21 in Iran, 2 in Turkey, and 1 in Thailand). This geographical concentration likely reflects a regional recognition of the importance of addressing female sexual health during pregnancy, particularly in societies where conservative cultural and religious norms have historically limited open discussions on the topic. Recently, however, there has been a growing awareness and academic engagement in these regions regarding women's sexual well‐being during pregnancy, a period highly valued in Islamic culture.^41^, ^42^

The increase in studies from 2010 onwards shows a shift towards recognizing sexual health as an integral part of maternal health in these regions. However, this geographical concentration introduces regional bias, which may limit the external validity of the findings.^41^, ^42^ These results reinforce the need for further research in diverse cultural and socio‐economic contexts to ensure that interventions like the PLISSIT model, CBT, and sexual education can be applied globally. Future research should aim to expand the geographical scope to include underrepresented regions, thereby enhancing the generalizability and robustness of the evidence base.

### 4.2 Comparison with existing literature

A systematic review conducted by Ribeiro et al.^43^ could not make clear and definitive recommendations for treating sexual dysfunction during pregnancy, as it included only two eligible articles. In contrast, the present meta‐analysis included studies on CBT, the PLISSIT model, and sexual education, all of which emphasize psychosocial aspects and use psychological strategies to improve sexual function. Ouyaba and Kesim^44^ identified psychological factors associated with sexual dysfunction during pregnancy. Consistent with their findings, systematic reviews by Rivera Felix et al.^45^ and Alizadeh et al.^46^ pointed out that pregnant women may experience sexual dysfunction as the result of psychosocial factors such as inaccurate beliefs or myths about sexual activity, cultural, religious, and social restrictions, and taboos, causing these women to avoid expressing their sexuality.

Additional studies have explored psychological factors and interventions. Brotto and Luria^47^ discussed how psychological aspects such as anxiety, body image, and cultural beliefs contribute to sexual dysfunction during pregnancy. Similarly, Alizadeh et al.^46^ emphasized the significant impact of cultural beliefs and attitudes on sexual function during pregnancy, reinforcing the importance of addressing these factors in treatment approaches. Moreover, Lutgendorf et al.^48^ found that cognitive‐behavioral interventions are effective in improving sexual function during pregnancy, which aligns with our findings on CBT. These studies further emphasize the importance of psychological interventions in addressing sexual issues during pregnancy.

Regarding other interventions evaluated, findings about Kegel exercises showed a significant increase in satisfaction and sexual function scores in the intervention groups. All studies evaluating mindfulness showed improvements in sexual function compared with placebo. For instance, Saniei et al.^35^ found no significant difference in sexual desire scores between treatment and control groups but noted an improvement in sexual satisfaction. Naji Abhary et al.^29^ presented significant results in all dimensions of sexual satisfaction. Similarly, Cengizhan and Uçar^22^ found significant improvement in attitudes towards sexuality in the mindfulness group compared with the control group. The RCT conducted by Yildiz Karaahmet et al.^39^ showed a significantly higher mean FSFI score for the yoga group compared with the control group. Finally, for PFME, the mean total sexual function score was significantly higher in the intervention group during pregnancy.^34^

### 4.3 Strengths and limitations

The strengths of this review include the comprehensive compilation of all non‐pharmacologic therapies for treating sexual dysfunction in pregnancy and the restriction to RCTs to reduce confounding factors and ensure better data quality and higher levels of scientific evidence. The rigorous search criteria, careful selection, and thorough assessment of article quality and scientific evidence, following Cochrane guidelines, also add to the strengths of this study.

The main limitation of our study is the heterogeneity in the design of the RCTs, especially in the follow‐up period post‐intervention. Another limitation is the low number of participants in some studies, which restricts the ability to draw robust conclusions from the data analysis. Therefore, interpretation of the results must be done with caution.

### 4.4 Implications

The available evidence provides promising results regarding the effectiveness of non‐pharmacologic therapies for treating sexual dysfunction during pregnancy. These findings may be especially useful for healthcare professionals involved in the care of pregnant women and provide scientific evidence for policy‐makers to promote quality public policies for this population group.

## 5 CONCLUSIONS

Our meta‐analysis showed that CBT, the PLISSIT model, and sexual education can improve sexual function during pregnancy. However, this review underscores the need for more studies with rigorous methodologies to increase the quality of evidence and better guide clinical practice.

## AUTHOR CONTRIBUTIONS

ACQA, ACAS and AKG designed the study. ACQA, ACZS, BBS, and HDM screened the abstracts for inclusion in the study. ACQA, ACZS and TBN analyzed the data. ACQA, ACAS, and AKG coordinated discussions and helped interpret the data. Risk of bias was assessed by ACQA, CLF and ACAS. The grade was assessed by ACQA, CLF and ACAS. ACQA, ACAS, CLF, MLF, and AKG drafted the manuscript, which was then critically reviewed by all authors. All authors approved the final manuscript.

## CONFLICT OF INTEREST STATEMENT

The authors have no conflicts of interest.

## Supporting information


Data S1.

Data S2.

## Data Availability

Research data are not shared.
