# Supplementary material for: Non‐pharmacologic therapies for treating sexual dysfunction during pregnancy: A systematic review and meta‐analysis
Source: Int J Gynaecol Obstet. 2025 Aug 27;172(3):1317–34. doi: 10.1002/ijgo.70451 (PMC12936644; doi:10.1002/ijgo.70451)
Supplement: Supplementary file 1 — Data S1. [file IJGO-172-1317-s002.pdf]

Peer Review of Electronic Search Strategies

***PRESS Guideline* — Search Submission & Peer Review Assessment**

SEARCH SUBMISSION: July 3, 2024

Searcher: Antonio Carlos Queiroz de Aquino	E-mail : carlos.queiroz.069@ufrn.edu.br
Date submitted: July 3, 2024	Date requested by: July 3, 2024

Systematic Review Title:

"Non-Pharmacological Therapies for Treating Sexual Dysfunction During Pregnancy: A Systematic Review and Meta-Analysis"

This search strategy is…

X	My PRIMARY (core) database strategy — First time submitting a strategy for search question and database
	My PRIMARY (core) strategy — Follow-up review NOT the first time submitting a strategy for search question and database. If this is a response to peer review, itemize the changes made to the review suggestions
	SECONDARY search strategy— First time submitting a strategy for search question and database
	SECONDARY search strategy — NOT the first time submitting a strategy for search question and database. If this is a response to peer review, itemize the changes made to the review suggestions

Database

(i.e., MEDLINE, CINAHL…): *[mandatory]*

PubMed/MEDLINE, ClinicalTrials.gov, Embase, Web of Science, PsycInfo, PEDro, Cochrane, and Scopus.
PubMed/MEDLINE will be used as the basis for validation of the strategy.

Interface

(i.e., Ovid, EBSCO…): *[mandatory]*

PubMed

Research Question

(Describe the purpose of the search) *[mandatory]*

What are non-pharmacological interventions to treat sexual dysfunction in pregnant women?

PICO Format

(Outline the PICOs for your question — i.e., Patient, Intervention, Comparison, Outcome, and Study Design — as applicable)

**P**	pregnant women with sexual dysfunction
**I**	non-pharmacological therapies used to treat pregnant women with sexual dysfunction
**C**	placebo or other treatment
**O**	improvement of sexual dysfunction
**S**	randomized clinical trials

Inclusion Criteria

(List criteria such as age groups, study designs, etc., to be included) *[optional]*

Randomized Clinical Trials (RCTs) that compared non-pharmacological interventions for treating sexual dysfunction during pregnancy were included.

Exclusion Criteria

(List criteria such as study designs, date limits, etc., to be excluded) *[optional]*

Cohort studies, systematic reviews, pilot studies, observational studies, under 18 years of age, studies that do not specifically sexual dysfunction in pregnant women. Studies that do not have a control group (pharmacological, non-pharmacological or placebo) Furthermore, studies that do not report the expected results will not be analyzed.

Was a search filter applied?

Yes **X**   No □

If YES, which one(s) (e.g., Cochrane RCT filter, PubMed Clinical Queries filter)? Provide the source if this is a published filter. *[mandatory if YES to previous question — textbox]*

In the EMBASE database, the filter for SEXUAL DYSFUNCTION was applied in the DISEASE option.

Other notes or comments you feel would be useful for the peer reviewer? ***[optional]***

None

Please copy and paste your search strategy here, exactly as run, including the number of hits per line. ***[mandatory]***

(Add more space, as necessary.)

	**MESH AND SYNONYM***	**STRATEGY LINES**	**NUMBER OF STUDIES LOCATED**
**P**	Pregnancy **(MeSH)**<br>Gestation<br>Pregnant Women **(MeSH)**<br>Women, Pregnant	(Pregnancy OR Gestation OR Pregnant Women OR Women, Pregnant)	-
	**AND**		
**I**	Therapeutic<br>Therapeutics **(MeSH)**<br>Therapy **(MeSH)**<br>Therapies<br>Treatment<br>Non-pharmacological treatment	(Therapeutic OR Therapeutics OR Therapy OR Therapies OR Treatment OR non-pharmacological treatment)	-
**C**	-	-	-
	**AND**		
**O**	Sexual Dysfunctions, Psychological **(MeSH)**<br>Physiological Sexual Dysfunction<br>Dysfunction, Psychological Sexual<br>Sexual Dysfunction, Physiological **(MeSH)**<br>Sexual Arousal Disorder<br>Sexual satisfaction<br>Sex Disorders<br>Sexual Distress<br>Body Image **(MeSH)**	(Sexual Dysfunctions, Psychological OR Physiological Sexual Dysfunction OR Dysfunction, Psychological Sexual OR Sexual Dysfunction, Physiological OR Sexual Arousal Disorder OR Sexual satisfaction OR Sex Disorders OR Sexual Distress OR Body Image)	-
**S**	-	-	-

*Use adaptations of the acronym as necessary.

DATABASES	STRATEGY*	NUMBER OF STUDIES LOCATED
PubMed/MEDLINE	(Pregnancy OR Gestation OR Pregnant Women OR Women, Pregnant) AND (Therapeutic OR Therapeutics OR Therapy OR Therapies OR Treatment OR non-pharmacological treatment) AND (Sexual Dysfunctions, Psychological OR Physiological Sexual Dysfunction OR Dysfunction, Psychological Sexual OR Sexual Arousal Disorder OR Sexual satisfaction OR Sexual Dysfunction, Physiological OR Sex Disorders OR Sexual Distress OR Body Image)	4276
Scopus	Pregnancy OR Gestation OR “Pregnant Women” OR “Women, Pregnant” AND Therapeutic OR Therapeutics OR Therapy OR Therapies OR Treatment OR “non-pharmacological treatment” AND “Sexual Dysfunctions, Psychological” OR “Physiological Sexual Dysfunction” OR “Dysfunction, Psychological Sexual” OR “Sexual Arousal Disorder” OR “Sexual satisfaction” OR “Sexual Dysfunction, Physiological” OR “Sex Disorders” OR “Sexual Distress” OR “Body Image”	611
Embase	(Pregnancy OR Gestation OR Pregnant Women OR Women, Pregnant) AND (Therapeutic OR Therapeutics OR Therapy OR Therapies OR Treatment OR non-pharmacological treatment) AND (Sexual Dysfunctions, Psychological OR Physiological Sexual Dysfunction OR Dysfunction, Psychological Sexual OR Sexual Arousal Disorder OR Sexual satisfaction OR Sexual Dysfunction, Physiological OR Sex Disorders OR Sexual Distress OR Body Image)<br>+ FILTER: DISEASES “SEXUAL DYSFUNCTION”	1052
Cochrane	(Pregnancy OR Gestation OR Pregnant Women OR Women, Pregnant) AND (Therapeutic OR Therapeutics OR Therapy OR Therapies OR Treatment OR non-pharmacological treatment) AND (Sexual Dysfunctions, Psychological OR Physiological Sexual Dysfunction OR Dysfunction, Psychological Sexual OR Sexual Arousal Disorder OR Sexual satisfaction OR Sexual Dysfunction, Physiological OR Sex Disorders OR Sexual Distress OR Body Image)	914
Web of Science	(Pregnancy OR Gestation OR Pregnant Women OR Women, Pregnant) AND (Therapeutic OR Therapeutics OR Therapy OR Therapies OR Treatment OR non-pharmacological treatment) AND (Sexual Dysfunctions, Psychological OR Physiological Sexual Dysfunction OR Dysfunction, Psychological Sexual OR Sexual Arousal Disorder OR Sexual satisfaction OR Sexual Dysfunction,	1822

	Physiological OR Sex Disorders OR Sexual Distress OR Body Image)	
ClinicalTrials.gov	“Sexual Dysfunction” AND (Pregnancy OR gestation OR “Pregnant women”)	273
PEDro	“Sexual Dysfunction” AND (Pregnancy OR gestation OR “Pregnant women”)	08
PsycINFO	“Sexual Dysfunction” AND (Pregnancy OR gestation OR “Pregnant women”)	61

PEER REVIEW ASSESSMENT: THIS SECTION TO BE FILLED IN BY THE REVIEWER

**Reviewer:** Adriana Alves da Silva Alves Dias / CRB-15/474	**E-mail:** biblioteca@ccs.ufrn.br	**Date completed:** 04/07/2024

**1. TRANSLATION**			
	A. No revisions	☑	
	B. Revision(s) suggested	☐	
	C. Revision(s) required	☐	

If “B” or “C,” please provide an explanation or example:

**2. BOOLEAN AND PROXIMITY OPERATORS**			
	A. No revisions	☑	
	B. Revision(s) suggested	☐	
	C. Revision(s) required	☐	

If “B” or “C,” please provide an explanation or example:

**3. SUBJECT HEADINGS**			
	A. No revisions	☑	
	B. Revision(s) suggested	☐	
	C. Revision(s) required	☐	

If “B” or “C,” please provide an explanation or example:

**4. TEXT WORD SEARCHING**			
	A. No revisions	☑	
	B. Revision(s) suggested	☐	
	C. Revision(s) required	☐	

If “B” or “C,” please provide an explanation or example:

5. SPELLING, SYNTAX, AND LINE NUMBERS			
	A. No revisions	☑	
	B. Revision(s) suggested	☐	
	C. Revision(s) required	☐	

If “B” or “C,” please provide an explanation or example:

6. LIMITS AND FILTERS			
	A. No revisions	☑	
	B. Revision(s) suggested	☐	
	C. Revision(s) required	☐	

If “B” or “C,” please provide an explanation or example:

7. OVERALL EVALUATION (Note: If one or more “revision required” is noted above, the response below must be “revisions required”.)			
	A. No revisions	☑	
	B. Revision(s) suggested	☐	
	C. Revision(s) required	☐	

Additional comments:

